# Apoptosis of Epithelial Cells and Macrophages due to Nonpigmented *Serratia marcescens* Strains

**DOI:** 10.1100/2012/679639

**Published:** 2012-05-02

**Authors:** Sylwia Krzymińska, Katarzyna Ochocka, Adam Kaznowski

**Affiliations:** Department of Microbiology, Faculty of Biology, A. Mickiewicz University, Ulica Umultowska 89, 61-614 Poznań, Poland

## Abstract

*Serratia marcescens* strains are opportunistic pathogens that are increasingly recognized as a cause of severe nosocomial infections. In this study we observed interactions between nonpigmented strains with human epithelial and macrophage-like cells. The strains revealed hemolytic activity only after the contact of the cells with erythrocytes. The contact of the bacteria with the host cells was also essential to their cytotoxicity. Moreover, all strains revealed adherence ability and were invasive to epithelial cells. Analyses of cellular morphology and DNA fragmentation of the HEp-2 and J774 cells exhibited typical features of cells undergoing apoptosis. We observed morphological changes, including condensation of nuclear chromatin and formation of membrane-bound apoptotic bodies. The lowest apoptotic index in HEp-2 cells did not exceed 25%, whereas the highest reached 59% at 24 h and 72% at 48 h after infection. Most of the strains (60%) induced fragmentation of nuclear DNA. The process depended on the activation of caspases, and was completely blocked by the pan-caspase inhibitor z-VAD-fmk. This study provided new insights into the mechanisms of nonpigmented *S. marcescens* pathogenesis. The results revealed that the strains produce cell-contact toxins that facilitate bacterial invasion, induce hemolysis, cytotoxicity, and apoptosis of host cells.

## 1. Introduction

*Serratia marcescens *is an opportunistic pathogen, increasingly recognized as a cause of morbidity in nosocomial settings. They are becoming an increasingly important cause of many outbreaks in neonatal intensive care units [1, 2]. The bacteria are frequent agents of catherization-associated infections. They are an etiological agent of wounds, urinary, and respiratory tract infections, keratitis, arthritis, meningitis, endocarditis, osteomyelitis, and septicemia [2].

Although there are frequent reports of nosocomial *Serratia* spp. outbreaks, the possible mechanism of their pathogenicity is still poorly understood and probably is complex and multifactorial. The strains secrete a number of toxins and exoenzymes: proteases, chitinases, a lipase, and nucleases, which may directly contribute to cellular cytotoxicity [2]. Some strains produce pore-forming toxins that represent hemolysin ShlA and ShlB. One of the most potent virulence factors of *Serratia* spp. is ShlA which causes hemolysis of human erythrocytes and the release of the inflammatory mediators from leucocytes. The toxin was shown to exhibit a cell-bound cytotoxicity [3]. Carbonell at al. [4] reported that *S. marcescens* strains produce an extracellular toxin that revealed cytotoxic activity to human epithelial cells. The toxin did not cause hemolysis of human erythrocytes.

Several bacterial pathogens have developed different strategies to survive inside the host, and they overcome natural defences and cause a disease. During infection, the first phase involves colonization of the wound surface, mucosal area or indwelling devices, next tissue infection, bacteraemia, and a systemic response may subsequently occur. Induction of apoptosis of the host cells has been considered to be a putative virulence mechanism that may cause tissue damage and facilitate further colonization [5].

*S. marcescens* strains associated with hospital outbreaks are mostly nonpigmented [2]. Despite considerable clinical data regarding their role in nosocomial infections, the pathogenic mechanism has not been elucidated. In this study we evaluated interactions between nonpigmented strains and human erythrocytes, epithelial cells, and murine macrophages originating from a well-established cell line, J774. 

## 2. Materials and Methods

### 2.1. Bacterial Strains

A total of 30 strains identified as *Serratia marcescens* by biochemical test kit API20E (bioMérieux) were used in this study. These strains were isolated from various specimens of hospitalized patients: 9 from urine (MPU S3, 12, 18, 21, 30, 33, 35, 36, and 37), 5 from postoperative wounds (MPU S6, 11, 26, 27, and 29) and 5 from ulcerations (MPU S1, 4, 7, 23, 42), 5 from secretions: aspirate, a conjunctival sac, and pus; from the pharynx, ear and drain (MPU S2, 28, 5, 14, and 13), 3 from intubation tubes (MPU S31, 34, and 41), and 1 from blood (MPU S22), stool (MPU S15), and a catheter (MPU S20). The isolates were maintained at −75°C in trypticase soy broth (TSB, Difco) containing 50% (vol/vol) glycerol. The HEp-2 and J774 monolayers were infected separately with an *E. coli* K-12 C600 strain as the negative control.

### 2.2. Cell Culture

The murine macrophage cell line, J774, was maintained in a growth medium (GM), containing RPMI 1640 (Biomed, Poland) supplemented with 10% heat-inactivated fetal calf serum (FCS, Gibco), gentamicin (5 mg mL^−1^), and 2 mM L-glutamine (Sigma). Human laryngeal epithelial cells (HEp-2) were cultured in a growth medium (GM), including the Minimum Essential Medium Eagle (MEM, Biomed) supplemented with 5% fetal calf serum, 2 mM glutamine, 80 IU penicillin per mL, 80 *μ*g streptomycin, and 1 mg mL^−1^ of nystatin. The cells were seeded with 100 mL of suspension in number of 2 × 10^6^/mL and incubated at 37°C in an atmosphere with 5% CO_2_ [6–8].

### 2.3. Infection Conditions

For each experiment, HEp-2 and J774 cells at the concentration of 2 × 10^6^/mL were seeded into 96-well plates (Nunc) and allowed to attach overnight. The strains were cultivated on Luria-Bertani agar (LB, Difco), harvested and resuspended in PBS to 1 on the McFarland scale, and diluted 1 : 100 in GM to a number of approximately 2 × 10^7^/mL. The aliquots were diluted in PBS and viable bacteria quantified. Bacteria cell suspension (100 *μ*L) was added to each well, to achieve a multiplicity of infection (MOI) of 10 (HEp-2 cells in the number of 2 × 10^6^ incubated with approximately of 2 × 10^7^ of bacteria) for 1 h at 37°C. Next, the medium was replaced with GM containing gentamicin (0.1 mg/mL) for 2 h at 37°C to kill extracellular bacteria. After three-time washing in PBS, the cells were incubated in the medium without gentamicin for 24 and 48 h.

For preparing bacterial filtrates, the strains were cultured in the Luria-Bertani medium in a shaking incubator with agitation at 300 rpm of 37°C for 24 h [8]. After centrifugation at 3000 ×g for 20 min, the supernatants were sterilized through 0.22 *μ*m-pore size filter membrane Millex-GV (Millipore).

### 2.4. Hemolytic Activity

The assay for contact-dependent and extracellular hemolysis was performed on a suspension of 1% human erythrocytes. Fresh human blood was obtained from volunteer donors from the Blood Donation Center. The blood was centrifuged (1500 ×g for 10 min), the plasma was discarded, and the erythrocytes were washed three times and resuspended in PBS to obtain a 1% (vol/vol) suspension. In order to investigate the possible presence of an extracellular hemolysin or factors responsible for hemolysis, the assay was also performed with a bacterial culture supernatant. The bacteria cell suspension or sterile culture supernatant was mixed with equal volume of 1% suspension of erythrocytes. The samples were centrifuged at 400 ×g for 10 min to allow close contact between bacterial cells and erythrocytes, next incubated at 37°C for 4 h. Then the samples were centrifuged at 1500 ×g for 10 min to remove unlysed cells. Hemolytic activity was expressed as percentage of total hemolysis, compared to 100% lysis in distilled water [9].

### 2.5. Cell-Contact Cytotoxicity

Cytotoxic activity of *S*.* marcescens* to HEp-2 cells was measured in the MTT (3-4,5-dimethylthiazol-2-yl-2,5diphenyltetrazolium bromide) assay and was done, as previously described [6]. The test assessed mitochondrial dehydrogenase activity as a marker of cytotoxicity. Briefly, the bacteria cells or culture supernatant (as described in section: Infection conditions) were directly added to the HEp-2 monolayer which was incubated for 4 hours. Next, they were removed, and the epithelial cells were washed with PBS, followed by addition of 200 *μ*L MTT and incubated for 4 h at 37°C. The medium was discarded, and the cells were lysed in a mixture of isopropanol:1 N HCl. Absorbance was measured in a microplate reader. Relative cytotoxicity was expressed as the percentage and calculated as follows:

(1) $$\%\;\;\text{cytotoxicity}=\left[1-\frac{{\text{OD}}_{\text{treated}\;\text{cells}}}{{\text{OD}}_{\text{untreated}\;\text{cells}}}\right]\times 100.$$

We used culture plates and tissue culture inserts (Nunc) with the anopore membrane with pore diameter of 0.2 *μ*m to test whether the contact with host cells is essential to *S. marcescens* cell cytotoxicity. HEp-2 cells were cultured in the lower chamber. The following day the bacteria cells at MOI of 10 were added in the upper chamber and incubated for 4 h. Assays were performed in triplicates in two separate experiments for each isolate.

### 2.6. Adhesion and Invasion of Epithelial Cells

*S. marcescens* adhesion and invasion to HEp-2 cells were quantified by a gentamicin survival assay based on the methods of Krzymińska et al. [7] with modifications. To avoid the cytotoxic effects of the strains, the epithelial cells were incubated with the bacteria at a MOI of 10 per cell. Infected cells were incubated with the bacteria for 2 h at 37°C. To determine the total number of cell-associated and intracellular bacteria, the monolayers were washed with PBS and lysed with 0.1% Triton X-100 in PBS. The total number of bacteria was determined by plating the lysates onto LB agar. To determine the number of internalized bacteria, infected HEp-2 cells after 2 h were treated with GM containing 0.1 mg/mL gentamicin to kill extracellular bacteria. After washing twice with PBS, the cells were lysed with Triton X-100, and the intracellular bacteria were determined by plating the lysates onto LB agar. The number of attached bacteria was determined by subtracting the number of intracellular bacteria following invasion from the total number of bacterial cells. The results were expressed as the adhesion index (AdI), that is, the mean number of associated (CFU) bacteria per 100 HEp-2 cells. The number of intracellular bacteria is presented as Invasion Index (InvI), that is, the percentage of number of internalized bacteria per 100 HEp-2 cells in compare with the number of adhering bacteria. As a control, an invasive strain of *Yersinia enterocolitica* O: 8/1B (pYV^+^) and nonpathogenic *E. coli* K-12 C600 were included. The data are presented as means from two independent experiments performed in triplicate.

### 2.7. Analysis of Apoptosis of Epithelial Cells and Macrophages

The infected cells in morphological assessment were stained with acridine orange (AO) and ethidium bromide (EB) and examined under laser confocal microscopy at 24 and 48 h after infection. Visible cells (green nuclei), apoptotic (fragmented red nuclei) with apoptotic bodies, and necrotic (structurally normal red nuclei) were quantified by counting 100 cells. We analysed DNA fragmentation as a biochemical marker of apoptosis. DNA from the infected cells was isolated as described previously [7]. At 24 and 48 h the cells were treated with a lysing buffer containing 100 mM NaCl, 10 mM Tris-HCl, 1 mM EDTA, 1% SDS pH 7.5, and 200 mg mL^−1^ proteinase K for 16 h at 37°C. After extraction with phenol-chloroform and precipitation with ethanol at −20°C, DNA was dried and dissolved in 10 *μ*L of TE buffer (10 mM Tris pH 8.0, 1 mM EDTA) and digested with 2 mg/mL of RNase. DNA fragmentation was observed after electrophoresis which was performed in 1.5% agarose gel (Basica LE GQT, Prona) at 120 V for 3 h. Gene Ruler 100 bp DNA Ladder (MBI Fermentas) was used as a molecular weight marker. DNA was stained with ethidium bromide, visualized under UV light, and digitalized with a Bio-Print V.99 system (Vilbert-Lourmat, France). 

### 2.8. Caspase Inhibition

To determine whether *S. marcescens*-apoptosis was dependent on caspases activation, we investigated inhibition of DNA fragmentation in HEp-2 and J774 cells by the broad-specificity caspase inhibitor N-benzyl-oxycarbonyl-Val-Ala-Asp(OMe)-fluoromethyl-ketone (z-VAD-fmk; R&D Systems). The inhibitor functions by forming a covalent bond with the cysteine in the active site of caspases [10]. The cells were pretreated with the inhibitor (100 *μ*M) for 90 min before infection with *S. marcescens* strains, next during the infection and for further 24 h [11].

### 2.9. Determination of Free Endotoxin Levels

We assessed if gentamicin treatment during invasion assay led to release of free bacterial LPS that could induce apoptosis of mammalian cells. Therefore, we measured free LPS levels in bacterial culture supernatant and the culture medium before and after 2 h of incubation with gentamicin. Free bacterial LPS was determined using the quantitative chromogenic *Limulus* Amoebocyte Lysate (LAL) test (Chromogenix AB, Coatest Endotoxin, Sweden) according to the manufacturer's instruction. Purified *E. coli* 0111:B4 reference endotoxin, bacterial cell suspensions, and culture media were dissolved in PBS, and serial dilutions were made. The samples were mixed with the LAL and chromogenic substrate reagents. The absorbance of the sample was determined spectrophotometrically at 405 nm, and the concentration of LPS was calculated from a standard curve. The amount of LPS was expressed in endotoxin units (EUs) per milliliter, as means ± S. D. of three replicates.

### 2.10. Statistical Analysis

The means and standard deviations of all results were calculated after performing the assay repeated on two independent experiments each in triplicate. A one-way analysis of variance ANOVA with Tukey's post hoc test at the significance level *P* < 0.05 was performed. The linear regression analysis was used to examine pairwise correlation between the Apoptotic Index, Invasion Index, cell-contact cytotoxicity, and contact-dependent hemolytic activity, and the Pearson correlation coefficient was determined. *P* < 0.05 were considered statistically significant. The statistical analysis was performed using Statistica PL software (StatSoft Poland Inc., USA).

## 3. Results

### 3.1. Cell-Contact and Extracellular Hemolytic Activity of *S. Marcescens* Strains

A quantitative assay was developed to characterize hemolytic activity of the strains. The results showed that *S. marcescens* cells were able to lyse human erythrocytes (Table 1). The hemolytic activity was ranging between 0.7% and 74.3%. The highest activity was observed for 8 (27%) strains. The lowest was revealed by 11 (37%) strains. Low percentage of hemolysis occurred with a bacterial culture supernatant. The highest extracellular hemolysin activity was in the range from 12.9 to 17.2% for 6 (20%) of the strains. Nonpathogenic *E. coli* K-12 C600 cells and culture supernatant did not reveal hemolytic activity.

### 3.2. *S. Marcescens* Cell-Contact Cytotoxicity

**Figure 1:**
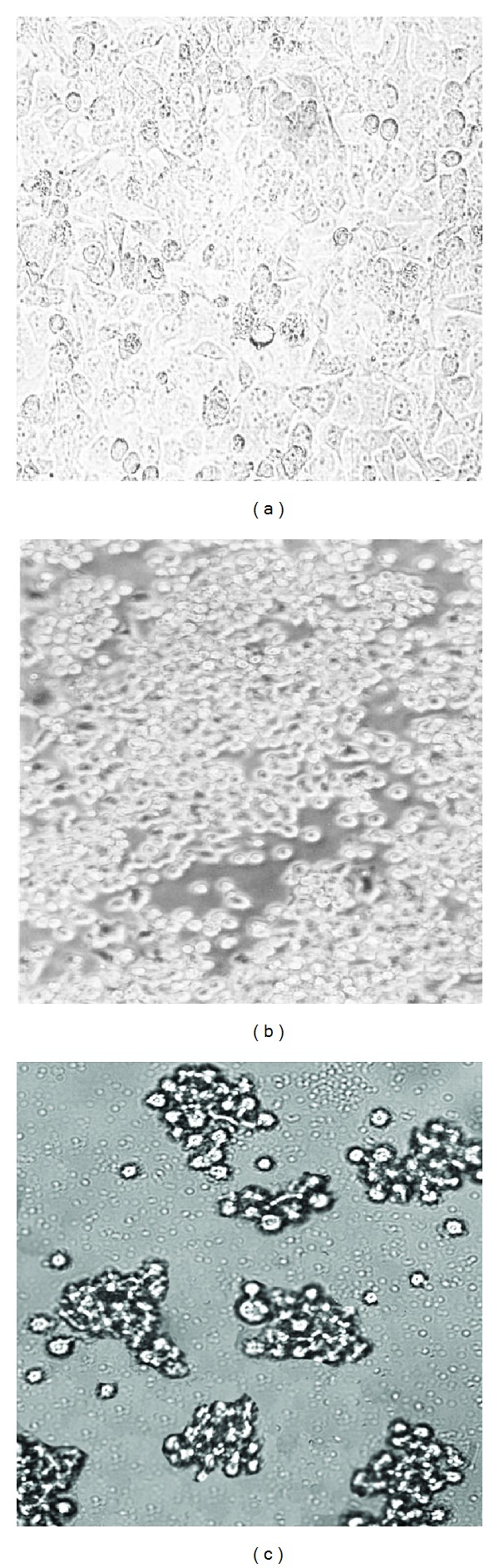
Cytotoxic effect of *S. marcescens* strains to HEp-2 cells. The monolayer was infected with an MPU S42 strain and observed using an inverted microscope, (a) at 1 h postinfection, (b) at 2 h p.i., and (c) at 4 h.

All live *S. marcescens* cells were found to be cytotoxic to epithelial cells after 4 h of infection. Microscopic examination of HEp-2 cells following incubation with bacterial cell suspension revealed a number of changes: rounding and shrinking of the cells, followed by detachment, loss of cytoplasmic extension, and disorganization of the cell monolayer. Using an inverted light microscope, we observed that epithelial cells lysis proceeded in a time-dependent manner (Figures 1(a)–1(c)). No cell lysis was observed at 1 h postinfection (Figure 1(a)). The cell lysis became apparent at 2 h (Figure 1(b)), and at 4 h the monolayer was in majority destroyed (Figure 1(c)). Moreover no cell lysis was observed at 4 h p.i. with nonpathogenic *E. coli* K-12 C600 strain. The suspensions of 13 (43%) bacterial cells showed the highest cytotoxicity percentage more than 52% as determined by the MTT assay, while 7 strains (23%) revealed the lowest activity in the range from 3.7 to 9.1% (Table 1). Moreover *S. marcescens* cells were cytotoxic to macrophages. The highest activity, above 70%, was observed for 13 (43%) strains. No cytotoxicity could be noticed when *S. marcescens* cells were not allowed to contact with epithelial cells and macrophages in the culture inserts. Bacterial culture supernatants did not show any effect on viability of the cells at the same time of incubation, which suggested that a contact-dependent cytotoxic factor may contribute to the cytotoxicity. Negative control *E. coli* K-12 C600 cells did not induce cytotoxic effects.

### 3.3. Adhesion and Invasion of HEp-2 Cells by *S. Marcescens* Strains

The adhesion and invasion of HEp-2 cells were quantified by a gentamicin survival assay after a 2-h exposure period with the strains. All *S. marcescens* strains were gentamicin sensitive. All *S. marcescens* strains were gentamicin sensitive and unable to grow in media containing the antibiotic at concentration of 100 *μ*g/mL. Minimum inhibitory concentration (MIC) of the strains was 6.3 *μ*g/mL or less. The adhesion index (AdI) ranged from 11 × 10^5^ to 58 × 10^5^ CFU/100 HEp-2. All strains revealed higher efficiency of adhesion in compare with nonpathogenic control which had index of 0.15 × 10^4^ CFU. The adhesion level of invasive strain of *Y. enterocolitica* O: 8/1B elevated to 39 × 10^5^ CFU per mL.

All strains invaded of HEp-2 cells at a significantly greater value than *E. coli* K-12 C600, nonpathogenic control (*P* < 0.01). The percentage of associated bacteria that were internalized ranged from 1.7% to 59.8% (Table 1). Five strains (17%) showed the highest invasion activity. Eight isolates (27%) showed the lowest invasive ability (1.7–8.7%). The InvI for *E. coli* K-12 C600 strain was 0.002% of total adhered cells, whereas for *Y. enterocolitica* O: 8/1B reached 71.8%.

### 3.4. Effect of *S. Marcescens* Infection on Apoptosis of Epithelial Cells and Macrophages

**Figure 2:**
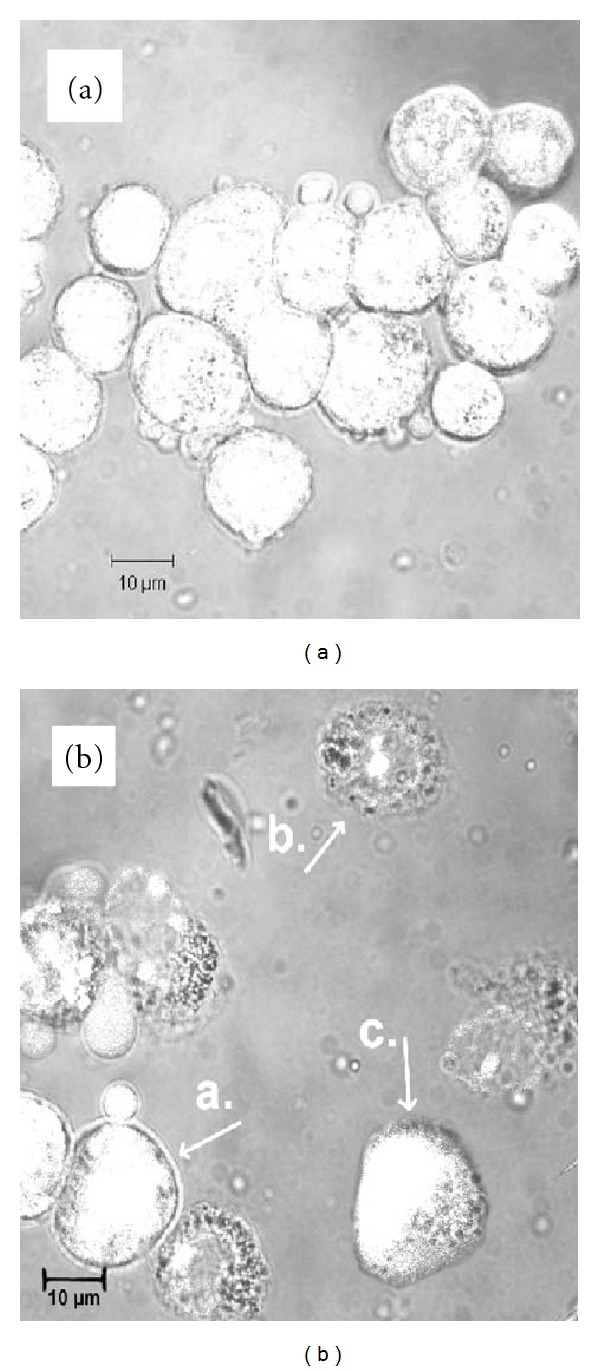
Apoptosis of HEp-2 cells. The cells were stained with ethidium bromide and acridine orange (100 *μ*g/mL) and observed in a laser confocal microscope. The cells were infected with (a) *E. coli* K-12 C600, (b) *S. marcescens* MPU S23 at 48 h. The arrows point to: a: live, b: apoptotic with apoptotic bodies, c: necrotic cells.

**Figure 3:**
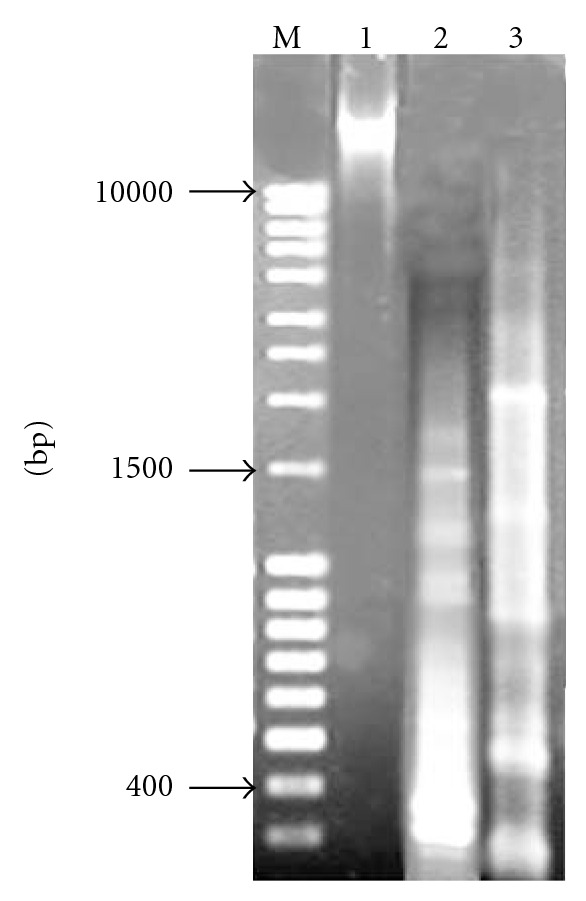
Analysis of intranucleosomal DNA fragmentation of HT29 cells incubated with different strains. M: molecular size marker. DNA from cells infected with Lane 1, strain of* E. coli* K-12 C600 (negative control). Lane 2, MPU S23 at 48 h after infection. Lane 3, MPU S29 at 48 h.

*S. marcescens *infection induced morphological changes of HEp-2 and J774 cells. Cell death was identified by their distinct morphology after 24 and 48 h of infection. It was observed as cellular shrinkage, round-up, and detachment from the culture plate. As shown in Figure 2, a remarkable difference in morphology was observed between the cells infected with nonpathogenic *E. coli* K-12 C600 (Figure 2(a)) and *S. marcescens* strains (Figure 2(b)). The apoptotic activity was expressed as the apoptotic index (AI), which was determined by ethidium bromide and acridine orange staining and observation under a laser confocal fluorescence microscope. Acridine orange permeates the cell membrane and makes the cells appear green. Ethidium bromide is only taken up by cells when cytoplasmic membrane integrity is lost, and stains nuclei red. Live cells had a normal green nucleus (Figure 2(a)), apoptotic cells displayed condensed or fragmented orange chromatin and membrane-bound apoptotic bodies (Figure 2(b)), the cells that died from necrosis were characterized by the loss of membrane integrity with a structurally normal red nucleus (Figure 2(b)). We found that all strains induced apoptosis to a different degree. The analysis of mean percentage of apoptotic cells at 24 h showed five groups with statistically significant differences between them (ANOVA, *F*_31,120_ = 155.31, *P* < 0.001). The highest apoptotic index ranging from 41.7% to 49.1% was observed for 6 (20%) strains. The lowest index (14.1–19.3%) was expressed by 2 (7%) strains. The percentage of apoptotic cells increased at 48 h. We found statistically significant differences between five groups of strains (ANOVA, *F*_31,  161_ = 167.713, *P* < 0.001). The highest apoptotic index (63.4–71.7%) was observed in cells infected with 10 (33%) strains. The lowest index (28.7–31.4%) was revealed by 3 (10%) strains. The mean apoptotic index of the nonpathogenic *E. coli* K-12 C600 strain was 5.1%  ± 1.2 at 48 h. Fragmentation of DNA into nucleosomal fragments, resulting in multimers of 180 to 200 bp, is one of the most distinctive biochemical features of apoptosis. The fragmented chromosomal DNA of the cells infected with *S. marcescens *strains was documented by the typical DNA ladder which was observed in HEp-2 cells infected with 18 (60%) strains at 48 h (Figure 3). The fragmentation was only observed when AI exceeded 53%.

Moreover, we observed morphological changes (cell shrinkage, chromatin condensation, and apoptotic bodies) of J774 cells upon *S. marcescens* infection. The highest apoptotic index ranging from 50.4 to 57.6% at 24 hours after infection was observed in phagocytes incubated with 6 (20%) strains (Table 1). Significantly the lowest AI, between 5.6 and 18.3%, was observed for 7 (23%) strains. The percentage of apoptotic cells increased at 48 h. The highest apoptotic index (59.1–65.3%) was observed in cells infected with 4 (13)% of the strains. The lowest index (25.7–31.9%) was revealed by 7 (23%) strains. The mean apoptotic index of the negative control was 3.4%  ± 1% at 48 h.

Some *S. marcescens* strains also caused necrosis of HEp-2 cells. The highest necrotic indexes (13.2–20.1%) were observed in epithelial cells infected with 5 (17%) strains at 24 h. The value of indexes increased to the range from 9.1 to 26.4%, for 6 (20%) strains at 48 h. Lower necrotic indexes were noted in macrophages infected with the strains and reached 6% and from 8 to 16% at 24 and 48 h, respectively.

*S. marcescens*-induced apoptosis was inhibited by addition of the pan-caspase inhibitor; thus caspases were involved in the process. The apoptotic indexes were reduced to the range between 4.9 and 9.1% after the treatment of HEp-2 and J774 monolayer with the inhibitor prior to the infection.

### 3.5. Free Endotoxin Levels

The assay was done for *S. marcescens* strain MPU S42 that expressed the highest AI value. There was no difference between free LPS concentration in culture medium containing bacterial cells before and after 2 h of incubation with gentamicin (1.4 ± 0.4 EU/mL) which suggested that gentamicin treatment did not induce release of significant amount of free LPS contributing to apoptosis of HEp-2 and J774 cells.

## 4. Discussion

Although nonpigmented strains of *S. marcescens *are an important cause of nosocomial infections, there is still little known about their mechanism of pathogenicity.

A common strategy used by bacterial pathogens is to secrete toxins and other factors to modulate the activity of host cells. In this study we observed that *S. marcescens *strains caused contact-hemolysis. The highest hemolytic activity was observed for strains isolated from urine (MPU S12, 30, 33), postoperative wounds (MPU S6, 29), intubation tubes (MPU S34, 31), and ulceration (MPU S42). Low hemolytic activity was observed in bacteria culture supernatants. The data suggested that the strains may produce cell-associated and -extracellular toxins. The major portal of pathogen entry are the epithelial skin layer and the layers coating the gastrointestinal, respiratory, and urogenital tracts. The epithelial host tissue is considered as an integral component of the mucosal immune system [12]. We observed that the contact of the bacteria with epithelial cells was essential to their cytotoxicity. The contact with six strains (MPU S6, 29, 31, 33, 34, and 42) induced the highest cytotoxic effect on epithelial cells after 4 h incubation. Moreover, the strains caused above 70% destruction of macrophage monolayer within after 4 h of the exposure to live cells. Supernatants from bacterial cultures tested in the same conditions had little or no cytotoxic activity. The results suggested that efficient lysis of erythrocytes, epithelial cells, and macrophages could be dependent on cell-bound or extracellular toxins produced by *S. marcescens* strains. Pore-formation mediated cytolysis of host cells is a strategy exploited by some pathogens to kill the cells. Hertle [3] suggested that *S. marcescens* strains produce hemolysin (ShlA) which represents the prototype of a new type of hemolysin family. The toxin exhibited a cell-bound activity that is not only a hemolytic, but also a cytolytic which damages the tissue and causes the release of the inflammatory mediators. The toxin is inactive on endothelial cells, but highly toxic to epithelial cells and may exert its cytotoxicity in direct contact of the bacteria with the host cells. Lin et al. [13] suggested that ShlA play a dominant role in *S. marcescens*-mediated infection model in rat.

The interaction of pathogenic bacteria with epithelial cells is the first stage of successive bacterial invasion of the host [14]. The ability to invade host cells is an important virulence factor. In our study, all strains were invasive with index higher than that of nonpathogenic control. The highest invasion index comparable to that of *Y. enterocolitica* O: 8/1B was observed for 5 (17%) *S. marcescens* strains, originated from ucleration (MPU S42), postoperative wounds (MPU S29, 6), and intubation tubes (MPU S34, 41). The Pearson linear correlation test revealed positive correlations between cell-contact hemolysis and Invasion Index (*r* = 0.52, *P* < 0.01). The results suggested that cell-bound hemolysins produced by the bacteria could be associated with invasion of epithelial cells. Hertle [3] suggested that *S. marcescens* pore-forming hemolysins play critical role in bacterial invasion of eucaryotic epithelial cells. Little is known about mechanisms of the process. Galindo et al. [15] suggested that *Aeromonas sobria* hemolysin activated production of intracellular Ca^+2^ and cAMP in epithelial cells. These changes might enhance bacterial invasion [16].

There is growing evidence that apoptosis of the host cells plays an important role in modulating the pathogenesis of a variety of infectious diseases. We examined interactions of *S. marcescens* strains and epithelial cells and demonstrated that one of the consequences of bacterial cytotoxicity is injury to the HEp-2 cells and cell death. The morphology of infected cells during the development of apoptosis is associated with compaction and margination of nuclear chromatin, condensation of the cytoplasm, and partition of the nucleus and cytoplasm into membrane bound-vesicles. Furthermore, the fragmentation in the internucleosomal linker regions, in multiple units containing 180–200 bp of the nuclear DNA, is a biochemical feature of apoptosis. At 48 h the highest apoptotic activity was expressed by 13 (43%) strains. A high level of cell-contact cytotoxic activity was consistent with the ability to induce HEp-2 cell death. The Pearson linear correlation test revealed positive correlations between the Apoptotic Index of infected HEp-2 cells at 24 h, cell-contact cytotoxicity (*r* = 0.59, *P* < 0.01), and cell-contact hemolytic activity (*r* = 0.77, *P* < 0.01) and the Invasion Index (*r* = 0.63, *P* < 0.01). The results indicated that *S. marcescens* strains can produce cell-bound cytotoxins that increase ability to invade epithelial cells and induced their apoptosis. A number of microbial factors that can trigger the induction of apoptosis of host cells have been identified. Previously, Carbonell et al. [4] observed that a cytotoxic toxin isolated from clinical strain was bound to the CHO cell surface, without being internalized and next was able to trigger changes in intracellular metabolism of the cells and to induce cell death by apoptosis. Hertle [3] suggested that ShlA induced irreversible vacuolation with subsequent lysis of epithelial cell lines and erythrocytes. Massive disruption of the host cell membrane by pore-forming toxins disturbed cellular homeostasis and resulted in cell death by apoptosis [5]. In the present study we observed that 9 (30%) strains induced high Apoptotic Index of epithelial cells above 60% and low cell-contact activity. High apoptotic activity could be associated with extracellular toxins produced by the strains. We have previously observed that bacterial culture supernatant 9 of 20 *S. marcescens* strains used in this study revealed cytotoxic activity to HEp-2 cells at 24 h incubation [8]. Previous studies suggested that *S. marcescens* strains produce RNase that display potent cytotoxic activity [17]. Shimuta et al. [9] reported that *S. marcescens* strains produced extracellular phospholipase A (PhlA) that revealed hemolytic and cytotoxic activity. PhlA induced destabilization of target cell membranes by directly hydrolyzing their phsphlipids. The pore induced by the toxins results in increasing cytosolic Ca^+2^ which could be the signal for the initiation of apoptosis. Carbonell et al. [4] isolated extracellular cytotoxic enterotoxin from a clinical isolate of *S. marcescens *which was highly cytotoxic to CHO cells but did not reveal hemolytic activity, which suggested that the cytotoxin is distinct from *S. marcescens* hemolysins.

Phagocytes, either resident in tissues or circulating in blood, contribute to the primary line of innate defence against bacterial pathogens by providing their removal and destruction at the level of epithelial barrier [5]. Some bacterial pathogens induce the death of immune cells to subvert normal host defence mechanisms, for invasion to deeper layers of the tissue. In the study we have demonstrated that macrophages originated from the murine J774 line were killed by infection with 3 and 8 of 30 *S. marcescens* strains at 24 and 48 h, respectively. In contrast, infection with nonpathogenic *E. coli* K-12 C600 and strains with low cytotoxic activity did not trigger apoptosis. The results suggest that the cell-contact and extracellular cytotoxins produced by the strains may suppress the epithelial and innate host immune cells, killing them through apoptosis. It could be a relevant mechanism of the bacteria to escape from the attack by the cells. Labbé and Saleh [18] suggested that killing of phagocytes impairs pathogen clearance and is detrimental to the host. So far, there has been no evidence about the signaling mechanisms of host cell apoptosis due to nonpigmented *S. marcescens* strains. Montaner et al. [19] observed that the culture supernatant of environmental isolate was responsible for apoptosis of cancer cell lines. The strain produced tripyrrole red-pigment, prodigiosin (PG), that is the reference compound of a family of drugs with potential application in cancer chemotherapy [20]. Grimont and Grimont [2] suggested that red-pigmented *S. marcescens* strains are predominantly associated with environmental settings, whereas the strains isolated from hospital outbreaks are mostly nonpigmented. Soto-Cerrato et al. [21] have suggested that the apoptotic signals are integrated at a mitochondrial level with releasing of proapoptotic cytochrome c to the cytosol, indicating that outer mitochondrial membrane permeabilization is an event in PG-induced apoptosis. Moreover, the expression of pro-apoptotic Bax protein and activation of caspases-9, -8, and -7 was observed in the cells [21].

We observed that infection with *S. marcescens* strains caused necrosis of HEp-2 and J774 cells. The highest necrotic index was observed for the cells infected with strains isolated from urine and ulcerations. It has been reported that the strains were the most common Gram-negative bacteria isolated as an etiologic agent of the contact lens related to microbial keratitis provoked by the necrosis of the cornea. These are due to one or more extracellular proteases produced by the strains. Soto-Cerrato et al. [21] examined the 56-kDa metalloprotease and found it to be the most potent cytotoxic factor that correlate with ulcerations of the cornea and tissue destruction.

The results of the study lead to a better understanding of nonpigmented *S. marcescens* pathogenesis. We demonstrate for the first time that the cell-contact pore-forming toxins produced by the bacteria facilitate invasion and induce hemolysis, cytotoxicity, and apoptosis of host cells. The process was mediated by the activation of the caspase pathway. It could be a strategy of the strains which contributes to the cellular damage to invade deeper mucosal layers and for a prolonged bacterial colonization.

## Figures and Tables

**Table 1:** Apoptotic index of HEp-2 and J774 cells infected with* S. marcescens *strains at 24 h and 48 h, cell-contact and extracellular hemolytic activity, cell-contact cytotoxic activity, and invasion ability.

Strain no.	Apoptotic index^1^ (HEp-2)	Apoptotic index (J774)	Hemolytic activity^2^		Cell-contact cytotoxicity^3^ [%]	Invasion index^4^ [%]
			Cell contact	Extracellular		
MPU S42	49.1/71.7	57.6/75.3	74.3	0.95	79.1	57.3
MPU S29	48.1/70.6	53.7/74.3	71.3	7.4	76.8	59.8
MPU S6	47.9/69.1	39.7/55.1	61.2	10.7	69.1	53.2
MPU S1	54.3/68.4	6.8/25.7	0.98	16.8	14.8	13.4
MPU S3	44.7/64.1	5.6/33.3	0.81	8.3	25.4	11.2
MPU S12	43.1/66.9	34.9/41.6	65.9	14.5	60.9	43.2
MPU S34	42.1/59.6	52.1/73.1	59.7	2.9	65.7	49.4
MPU S30	41.7/63.4	51.6/59.1	58.3	5.1	60.2	35.7
MPU S31	38.7/68.1	18.3/54.6	64.8	6.4	81.3	54.4
MPU S23	37.1/71.7	34.8/41.9	49.6	0.75	52.9	39.1
MPU S11	36.7/67.2	42.6/48.9	0.70	17.2	12.6	12.7
MPU S33	35.1/48.1	55.8/75.3	68.9	1.5	72.6	48.2
MPU S21	34.1/61.2	31.6/36.8	8.0	0.70	34.8	11.7
MPU S7	34.1/65.4	41.8/45.7	38.7	12.9	61.2	31.2
MPU S5	33.4/53.8	39.9/42.1	4.1	11.6	4.8	6.3
MPU S22	31.3/64.2	46.1/52.8	15.6	15.7	28.4	12.4
MPU S28	29.6/43.7	27.6/60.4	17.9	4.1	41.8	11.7
MPU S27	28.4/37.9	14.3/48.3	5.1	2.4	6.1	6.3
MPU S14	27.1/59.6	20.1/29.1	4.4	6.9	9.1	6.1
MPU S4	26.8/38.1	22.7/30.6	3.1	9.1	4.1	5.8
MPU S26	24.4/54.1	47.5/57.3	45.3	3.4	59.1	41.2
MPU S36	24.1/49.6	45.8/57.1	21.7	0.81	45.1	35.7
MPU S13	23.9/58.1	43.9/54.6	12.8	2.1	49.6	28.4
MPU S41	23.6/56.1	16.8/28.6	7.6	4.5	3.7	7.4
MPU S37	23.1/51.4	48.6/59.1	41.2	5.7	57.3	28.9
MPU S35	22.8/40.8	9.4/27.3	6.8	4.7	6.4	8.1
MPU S20	22.1/41.4	44.1/48.6	31.7	1.2	37.2	17.4
MPU S18	21.4/41.4	40.4/55.8	34.2	3.1	53.1	8.7
MPU S2	19.3/29.4	11.7/26.1	3.7	13.1	4.6	1.7
MPU S15	14.1/28.7	29.1/31.9	28.2	3.8	52.7	11.3

^1^The percentage mean of apoptotic cells at 24 and 48 h. ^2^The percentage of total hemolysis, compared to 100% lysis in distilled water was performed 4 h after infection by using a suspension of 1% human erythrocytes. ^3^The percentage of cytotoxicity was determined 4 h after infection by MTT assay. ^4^The percentage of number of internalized bacteria per 100 HEp-2 cells in compare with number of adhering bacteria. Values correspond to the means from two experiments in triplicate.
